# NK Cell-Based Immunotherapy in Renal Cell Carcinoma

**DOI:** 10.3390/cancers12020316

**Published:** 2020-01-29

**Authors:** Iñigo Terrén, Ane Orrantia, Idoia Mikelez-Alonso, Joana Vitallé, Olatz Zenarruzabeitia, Francisco Borrego

**Affiliations:** 1 Immunopathology Group, Biocruces Bizkaia Health Research Institute, 48903 Barakaldo, Spain; inigo.terrenmartinez@osakidetza.eus (I.T.); ane.orrantiarobles@osakidetza.eus (A.O.); imikelez@cicbiomagune.es (I.M.-A.); joana.vitalleandrade@osakidetza.eus (J.V.); olatz.zenarruzabeitiabelaustegui@osakidetza.eus (O.Z.); 2 CIC biomaGUNE, 20014 Donostia-San Sebastián, Spain; 3 Ikerbasque, Basque Foundation for Science, 48013 Bilbao, Spain

**Keywords:** NK cells, kidney cancer, renal cell carcinoma, IL-2, cancer immunotherapy, tumor microenvironment

## Abstract

Natural killer (NK) cells are cytotoxic lymphocytes that are able to kill tumor cells without prior sensitization. It has been shown that NK cells play a pivotal role in a variety of cancers, highlighting their relevance in tumor immunosurveillance. NK cell infiltration has been reported in renal cell carcinoma (RCC), the most frequent kidney cancer in adults, and their presence has been associated with patients’ survival. However, the role of NK cells in this disease is not yet fully understood. In this review, we summarize the biology of NK cells and the mechanisms through which they are able to recognize and kill tumor cells. Furthermore, we discuss the role that NK cells play in renal cell carcinoma, and review current strategies that are being used to boost and exploit their cytotoxic capabilities.

## 1. Introduction

Natural killer (NK) cells are large granular lymphocytes that were described more than 40 years ago [1,2]. They were initially characterized by their ability to kill cancer cells through, among others, an exocytosis mechanism of cytotoxic granules containing perforin and granzymes. Unlike cytotoxic CD8+ T cells, NK cells can directly induce cell death in the absence of prior sensitization. Over time, it has been described that besides killing tumor cells, they also kill virus-infected cells, and they have important cytotoxic activity against some healthy immune cells, such as activated T cells. In addition to their direct cytotoxic capacity, NK cells also produce cytokines as, for example, interferon gamma (IFNγ) and chemokines, such as the C-C motif chemokine ligand 3 (CCL3) and CCL4, after the ligation of activating receptors that are expressed on their surface and/or following the stimulation with several cytokines [3,4,5,6,7,8,9,10]. While NK cells are better known for their defense against viral infections and for surveillance against tumors, they are also appreciated for their participation in the generation of more efficient T helper type 1 (Th1) immunity, in the modulation of self-reactivity and of immune responses, in which their cytotoxic, as well as cytokine- and chemokine-producing capabilities have an important role [11,12,13]. Therefore, NK cells are currently considered to have a critical role in the maintenance of homeostasis and in the control of the immune response, promoting inflammation on the one hand, and restricting the adaptive immune response that could lead to excessive inflammation, and even autoimmunity, on the other. Furthermore, although NK cells have long been considered a part of the innate immune system, more recently have been described subpopulations of long-lived NK cells with effector functions characteristic of adaptive immunity [14,15,16,17,18].

## 2. NK Cell Development, Subsets, and Diversity 

NK cells constitute 5–15% of circulating lymphocytes and represent one of the three main human lymphocyte lineages, including T cells and B cells. There are resemblances between NK cells and T cells, mostly with CD8+ T cells [4]. Nevertheless, the developmental pathways of T cells and NK cells, how they detect tumor and infected cells, and the way they get activated are different. T cells develop in the thymus and are specifically activated when their T-cell receptor (TCR) recognizes foreign antigens in the context of major histocompatibility complex (MHC) molecules, called human leukocyte antigens (HLA) in humans [4]. In contrast, NK cells mainly develop outside the thymus, and do not express specific antigen receptors resulting from gene recombination, such as the TCR and B-cell receptor (BCR). NK cells’ effector functions are regulated by various types of activating and inhibitory receptors [10,14,19,20].

Currently, NK cells are classified as one of the main members of the family of innate lymphoid cells (ILCs), which are very important effector cells of the innate immune response [21,22]. ILCs respond immediately to infection and cellular damage, and also exert a very relevant influence on the development of the adaptive immune response through cytokine secretion and their cytotoxic activity. ILCs are classified into five subpopulations that could be considered the counterpart of effector T cells. Thus, ILC1 cells would be the counterpart of Th1 lymphocytes, ILC2 of Th2 lymphocytes, and ILC3 of Th17/Th22 lymphocytes. On the other hand, NK cells would be the counterpart of cytotoxic CD8+ T cells. ILCs also include the lymphoid tissue-inducer or LTi cells [21,22]. NK and ILC1 cells have a very similar phenotype, as well as similar effector functions, especially in relation to the pattern of cytokines that they secrete, which is mainly IFNγ. However, they differ in that ILC1 exhibits very little or no cytotoxic activity due to the low or zero levels of perforin and granzymes they express. In addition, they have a different expression pattern of transcription factors. NK cells require and express Eomes and T-bet transcription factors for their development, while ILC1 only express and require T-bet [21,22]. Moreover, with the exception of NK cells, many of which circulate, ILCs are primarily located in tissues.

Human NK cells are classically identified by the absence of TCR/CD3 and the presence of the CD56 molecule. Based on the intensity of CD56 receptor expression, NK cells are basically divided into two subpopulations: CD56^dim^ and CD56^bright^ [3,23,24]. The CD56^dim^ subset expresses low levels of the receptor, constitutes 90–95% of circulating NK cells, and is characterized by increased cytotoxic activity against targets and a lower capacity for cytokine production, such as IFNγ, in response to stimulation with interleukins such as IL-2, IL-12, IL-15, and IL-18. In addition, CD56^dim^ cells express the low affinity receptor for the Fc portion of immunoglobulin G (IgG) or FcγRIII, also called CD16, which is responsible for the antibody-dependent cellular cytotoxicity (ADCC). In contrast, CD56^bright^ cells express high levels of CD56, are the majority in secondary lymphoid tissues (SLT), have a lower cytotoxic capacity, and due to the low or null expression of CD16, also have less ADCC. On the contrary, they secrete higher levels of cytokines in response to the stimulation with interleukins [3,23,24]. In addition to the CD56^dim^ and CD56^bright^ NK cells, two more subsets have been described: CD56^neg^ and unconventional CD56^dim^ (unCD56^dim^) NK cells, the latter are characterized by the absence of CD16 expression [25]. These two subsets are present at very low frequencies in healthy donors and under homeostatic conditions. However, they are expanded in certain situations. For example, CD56^neg^ NK cells are expanded in human immunodeficiency virus (HIV)-infected patients with high viremia [26,27], and the unCD56^dim^ subset significantly increases in lymphopenic environments of patients after haploidentical hematopoietic stem cell transplantation (haplo-HSCT) [28,29]. Lastly, considering that CD56 is also expressed on ILC1 cells, more precise markers, such as NKp80, are also necessary to unequivocally identify NK cells [23].

Human NK cells develop from CD34+ hematopoietic stem cells (HSC) in the bone marrow [30]. These HSC differentiate first into lymphoid-primed multipotential progenitors (LMPP), which then become a common lymphoid progenitor (CLP). These CLPs further differentiate into NK cell progenitors (NKP) that are classified into three sequential stages of maturation, named NK cell progenitors (stage 1), pre-NK cells (stage 2), and immature NK cells (stage 3). The early stages of NK cell development and differentiation have been characterized in the context of the bone marrow niche, but pre-NK cells can be detected in the circulation, and other data have shown that they are enriched in extramedullary tissues where subpopulations of mature NK cells reside, suggesting that they have developed locally [3,25,31]. It is known that some NKPs are selectively enriched in SLT, such as lymph nodes and tonsils, as well as in the gastrointestinal tract, liver, and uterus [32,33,34,35,36,37]. The most accepted model of NK cell development occurs in the linear fashion just described above, in which the expression of CD94 marks the commitment to the CD56^bright^ stage (stage 4), that next differentiate into CD56^dim^ NK cells (stages 5 and 6) [25,31]. The differentiation into adaptive (also called memory) CD56^dim^ NK cells could subsequently occur after viral infection, as, for example, the human cytomegalovirus [25]. The CD56^neg^ cells are probably exhausted CD56^dim^ NK cells, although this has not been well-proven yet [25]. Related to the unCD56^dim^ NK cells, it has been suggested that they are an intermediate stage of differentiation between CD56^bright^ and CD56^dim^ NK cells [25]. The support for the linear model comes from analysis of NK cells in SLT and in vitro studies. However, more recent evidence also suggests the existence of a branched model in which different precursor populations can develop independently in distinct subsets of mature NK cells, that is, CD56^bright^, CD56^dim^, and adaptive NK cells [38].

**Figure 1:**
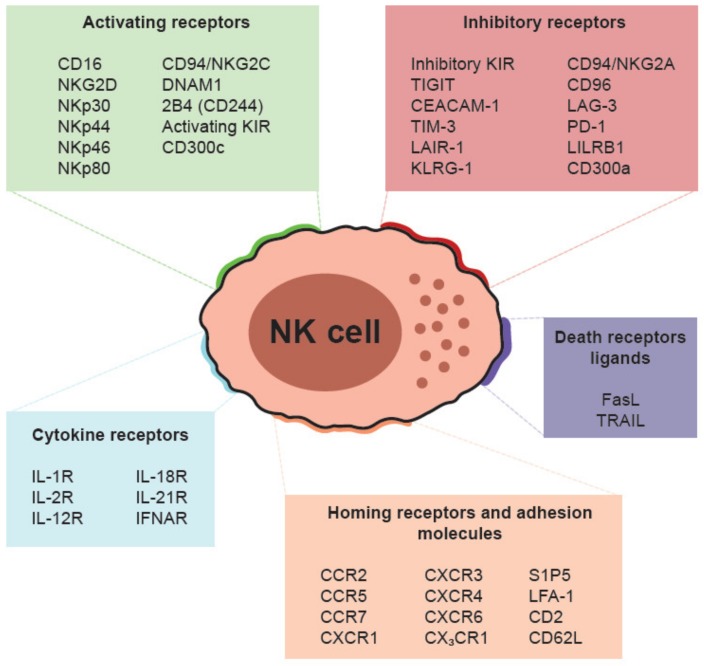
Surface receptor repertoire of human natural killer (NK) cells. DNAM1: DNAX accessory molecule 1. KIR: Killer-cell immunoglobulin-like receptors. TIGIT: T-cell immunoreceptor with Ig and ITIM domains. CEACAM-1: Carcinoembryonic antigen-related cell adhesion molecule 1. TIM-3: T-cell immunoglobulin and mucin-domain containing 3. LAIR-1: Leukocyte-associated immunoglobulin-like receptor 1. KLRG-1: Killer cell lectin-like receptor subfamily G member 1. LAG-3: Lymphocyte activation gene 3. PD-1: Programmed cell death protein 1. LILRB1: Leukocyte immunoglobulin-like receptor subfamily B member 1. FasL: First apoptosis signal ligand. TRAIL: Tumor necrosis factor-related apoptosis-inducing ligand. CCR: C-C chemokine receptor. CXCR: C-X-C chemokine receptor. CX3CR: CX3C chemokine receptor. S1P5: Sphingosine-1-Phosphate receptor 5. LFA-1: Lymphocyte function-associated antigen 1.

NK cells express a wide range of receptors on their surface, some of which are quite cell-specific in their expression [3,14,19,20,23]. Among them, NK cells express activating and inhibitory receptors, death receptor ligands, cytokine receptors, and homing and adhesion molecules (Figure 1). Some of the cell surface receptors are associated with developmental stages. For example, CD94/NKG2A is expressed on all CD56^bright^ NK cells (stage 4) and in a subset of CD56^dim^ NK cells in stage 5, while CD57 is a surface marker of replicative senescence and terminally differentiated CD56^dim^ NK cells in stage 6 [23,25]. Here, it is very important to note that research performed in recent years have established that in each individual and in any tissue, the population of NK cells is much more diverse than previously appreciated in terms of developmental, phenotypic, and functional parameters. In fact, it is clear that the traditional view of the NK cell lineage as a population of cells with very few subsets (i.e., CD56^bright^ and CD56^dim^), and relatively similar functions, is not entirely accurate. On the contrary, the NK cell lineage is remarkably diverse [23,39]. New technological approaches are helping the scientific community to characterize the NK cell lineage in depth. For example, by simultaneously analyzing 37 parameters on peripheral blood NK cells, an extraordinary degree of diversity was revealed, with there being an estimated 6,000 to 30,000 NK cell subsets within a given individual [40]. Also, single cell transcriptomics studies have revealed tissue-specific gene signatures that allow identification of NK cell populations that differ between tissues [41,42].

## 3. Cell Surface Receptors and Cytotoxic Mechanisms

NK cells induce cell death primarily through two different mechanisms. The most-studied route is degranulation, through which they release cytotoxic granules containing the pore-forming molecule perforin and death-inducing enzymes, such as granzymes, when activated against the target cell [43,44,45]. This pathway is triggered by activation signals from cell surface receptors. Other routes by which NK cells can kill target cells are the death receptors’ pathways: the tumor necrosis factor (TNF)-related apoptosis-inducing ligand (TRAIL)-TRAIL receptor (TRAILR), and the first apoptosis signal (FAS)-FAS ligand (FASL), also known as the CD95-CD95L pathway. Instead of triggering the release of cytotoxic granules, death receptor pathways induce apoptosis through the activation of caspases within the target cell leading to cytotoxicity regardless of activating receptor-mediated signals that control NK cell degranulation [46,47,48].

NK cell degranulation and the subsequent killing of the target cell is a very well-regulated process, so that the lytic granules are transported to the interface formed with the target cell and their contents (perforin and granzymes) are secreted in it. To carry this out, it is required for the NK cell to be in contact with the target cell, forming an immunological synapse [49,50,51,52]. This is a complex and very dynamic three-dimensional structure with intense activity of biochemical signals between the cells. Numerous molecules that participate in the immunological synapse have been identified, including surface receptors, signaling molecules, cytoskeleton elements, and cellular organelles [49,50,51]. The formation of a lytic immunological synapse of NK cells involves many stages that occur in a linear manner, in order to guarantee the secretion of perforin and granzymes present in the lytic granules towards the place of contact between the target cell and the NK cell, thus avoiding possible damage to the healthy cells that may be in the vicinity [49,50].

Moreover, NK cells can detect antibody-coated cells through CD16, thereby exerting ADCC and cytokine secretion. CD16 is coupled to the signal transduction polypeptides, also called adaptor proteins, CD3ζ and FcRγ, that contain ITAMs (immunoreceptor tyrosine-based activation motifs) [53,54]. In addition to ADCC, NK cells exert natural (direct) cytotoxicity against target cells in the absence of antibodies. Natural cytotoxicity receptors, or NCRs (NKp46, NKp44, and NKp30) are also potent activating receptors linked to the adaptor proteins CD3ζ, FcRγ, or DAP12 [55]. Other activation receptors include the NKG2D homodimer (which is associated with DAP10), DNAM1, 2B4 (CD244), CD94/NKG2C, CD300c, and so forth [14,56,57,58]. A characteristic of several NK cell-activating receptors lies in their ability to detect molecules induced under conditions of cellular stress [59,60]. This is the case of the NKG2D receptor, which interacts with several ligands (MICA, MICB, and ULBP1-6), which are not expressed or do so at very low levels in most tissues, but which are overexpressed as a consequence of cellular stress, such as during the DNA damage response [61]. Another example is the B7-H6, an NKp30 receptor ligand, which is not detected in healthy cells but is expressed in certain tumor cells [62].

In the mid-1980s, Kärre and his colleagues pioneered the “missing self” hypothesis, which describes the lack of expression of MHC class I molecules (self) in target cells as the common element that determines their susceptibility to NK cell-mediated lysis [10,63]. Loss of expression of MHC class I molecules can occur when cells are altered by viral infection or malignant transformation, thus being susceptible to NK cell killing. In contrast, healthy cells, by expressing MHC class I molecules, are protected from NK cells’ lysis. The recognition of the "missing self" is explained by the expression on the NK cell surface of a variety of specific inhibitory receptors for MHC class I molecules. These receptors include the polygenic and highly polymorphic family of inhibitory KIRs (killer-cell immunoglobulin-like receptors) in humans, Ly49 lectin molecules in mice, and CD94/NKG2A in both species [10,14,19,64,65]. In humans, while the CD94/NKG2A heterodimeric receptor binds to the HLA-E molecule, a non-classical HLA class I molecule, KIRs do bind to the classic HLA-A, -B, and -C molecules [19]. These MHC class I specific inhibitory receptors possess a long intracellular tail with one or more immunoreceptor tyrosine-based inhibitory motifs (ITIMs) that are responsible for the transmission of the inhibitory signal [66,67]. Certain subpopulations of NK cells also express the LILRB1 inhibitor receptor, also known as ILT2 and CD85j, whose ligands are a subgroup of HLA class I molecules [14]. In addition to these MHC class I specific receptors, there are other inhibitory receptors expressed by NK cells, such as TIGIT, LAIR-1, CD300a, and so forth [14,27,68].

**Figure 2:**
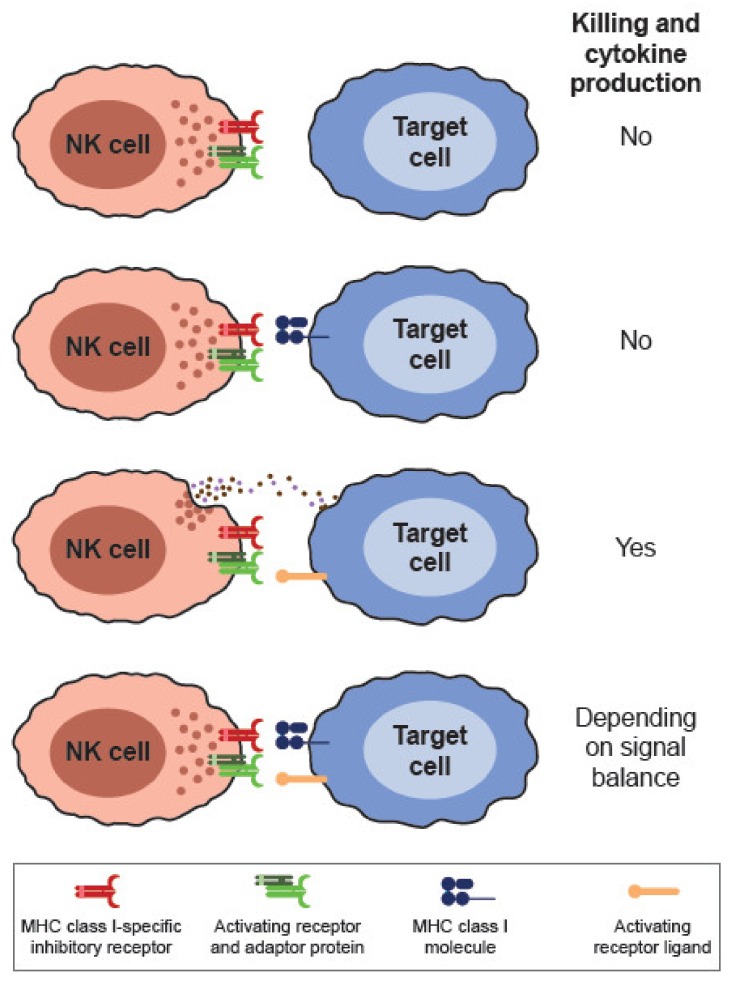
Activating and inhibitory signaling balance determines NK cell killing and cytokine production. Target cells expressing ligands for activating receptors trigger NK activation. When target cells also express ligands for inhibitory receptors, NK cell activation will depend on the signal balance.

The preservation or elimination of target cells with the consequent production of cytokines and chemokines will therefore depend on the result of the integration of activating and inhibitory signals originating from the NK cell surface receptors [20]. NK cells do not lyse healthy cells expressing MHC class I molecules and/or low or null expression of stress-induced molecules and other activating receptor ligands. On the contrary, they selectively kill target cells that have low levels of MHC class I expression and/or are expressing adequate levels of stress-induced molecules, such as NKG2D ligands, and other ligands for activating receptors [57,62] (Figure 2).

In addition to the important role of NK cells in the development of an adequate immune response against tumors and pathogens, it is also essential to maintain tolerance towards the host. This is achieved through a process called education or licensing, which is governed by the interaction of inhibitory receptors (KIR, Ly49, and CD94/NKG2A) with their ligands, the MHC class I molecules, during NK cell development [69,70,71,72,73]. Education could be defined as the process through which a NK cell is programmed to exert its effector functions and is calibrated to be inhibited by its own MHC class I molecules. In general terms, the education of a NK cell by a specific MHC class I molecule is defined by its ability to detect the decrease of that MHC class I molecule in a target cell that can be lysed [69,70,73]. In humans, up to 15 genes on chromosome 19 encode for KIR receptors. *KIR* genes are grouped into haplotypes and expressed in a stochastic manner, so that in a given individual there are various subpopulations of NK cells according to the number of KIR receptors they express [74,75]. Therefore, within a given repertoire, an individual can have educated NK cells, that is, those that during their development have interacted with their own MHC class I molecules, as well as uneducated NK cells, which are those that during their development have not interacted with MHC class I molecules [69,70].

## 4. NK Cells in Cancer Immunotherapy

More than 15 years have passed since the introduction of the pioneering works that established the potential of NK cells to mediate tumor regression. These studies demonstrated that NK cells from a haploidentical donor can prevent relapse after haplo-HSCT and also are able to induce remission after infusion of mature NK cells in patients with acute myeloid leukemia (AML) [76,77].

Several cytokines are currently being used in humans in terms of their ability to stimulate NK cell activity, at least partially, against tumors. Recombinant IL-2 was the first cytokine tested to stimulate the immune response in cancer patients [78,79,80]. Although early studies established the proof of concept of the therapeutic anti-tumor potential of IL-2, the responses were limited and its toxicity was substantial when used at high doses [81]. Later on, it was shown that a low dose of IL-2 had a lower toxicity profile, and it has been incorporated into an increasing number of assays to induce in vivo expansion and persistence of effector cells, such as NK cells, during adoptive cell therapy [77,82]. However, it should be noted that the use of low doses of IL-2 can also stimulate and expand regulatory T (Treg) cells, which suppress, among others, the proliferation and cytotoxicity of NK cells [83]. New variants of IL-2, such as those that selectively bind to the β-subunit of the IL-2 receptor (IL-2Rβ) expressed on NK cells, rather than the IL-2Rα subunit expressed in Treg cells, could provide better results [79,84,85]. IL-15 selectively stimulates CD8+ T cells and NK cells and prevents undesirable mobilization of Treg cells [86,87]. The first clinical trial with single-chain IL-15 (scIL-15) in cancer patients exhibited high dose-dependent toxicity [88]. Nevertheless, when used after the adoptive infusion of NK cells in patients with AML, scIL-15 promoted the persistence and proliferation of NK cells [80,89]. Importantly, IL-15 superagonists are being developed. An example is ALT-803, a complex consisting of a homodimer of mutated IL-15 linked to a fusion protein formed by the α-chain of IL-15R (IL-15Rα) and the Fc fragment of IgG1 [90,91]. ALT-803 has better pharmacokinetic properties, a longer half-life in lymphoid tissues, and importantly, has greater anti-tumor activity compared to scIL-15 [92]. Other than cytokines, there are several drugs that can directly and/or indirectly increase NK cell function in vivo. For example, lenalidomide indirectly increases the cytotoxicity and proliferation of NK cells through the release of IL-2 and IFNγ from surrounding T cells and the production of cytokines by dendritic cells [80,93].

Immune checkpoint inhibitors provide a blockade of inhibitory receptors [94]. PD-1 (programmed cell death protein 1) is expressed in activated T cells and NK cells [95], and along with its ligand PD-L1, has a central role in tumor recurrence and progression, since signaling through this pathway suppresses lymphocytes, including NK cells [80,95]. In vitro and in vivo experiments have shown that PD-1 and PD-L1 blockades elicit a strong NK cell response that is required for the full effect of the immunotherapy [80,96,97]. PD-1 blockade also increases ADCC mediated by NK cells and improves their traffic to tumors [80,97]. In addition, NK cells are able to express PD-L1, and it has been shown that the anti-PD-L1 monoclonal antibody (mAb) acts on PD-L1+ NK cells against PD-L1- tumor cells [98]. Other checkpoints that are mostly expressed in NK cells include, among others, KIR, CD94/NKG2A, and TIGIT [14,19,64,99,100]. Preclinical studies and clinical trials are currently studying the efficacy of the blockade of these checkpoints [94]. For example, anti-KIR mAbs increase tumor cell lysis mediated by NK cells and enhance ADCC in vitro [101,102]. Also, there are several clinical trials in phase I/II that have been completed or are still recruiting patients with anti-KIR mAbs, alone or in combination with other checkpoint inhibitors [94,103,104,105,106]. Related to CD94/NKG2A, it has been demonstrated that blocking its expression by means of a single-chain variable fragment derived from an anti-NKG2A Ab linked to endoplasmic reticulum retention domains overcomes (HLA-E+) tumor resistance to NK cells [107]. Furthermore, it has been demonstrated that the anti-NKG2A mAb monalizumab stimulates anti-tumor immunity by promoting NK cells and CD8+ T cells effector functions [108]. Tumor-associated NK cells also exhibit high expression levels of the checkpoint inhibitory receptor TIGIT, and mAb-mediated blockade of this receptor prevents NK cell exhaustion and elicits potent anti-tumor immunity [109]. Several clinical trials are going on, testing the safety and efficacy of anti-TIGIT mAbs alone or in combination with other mAbs [94].

Antibodies have also been used to direct NK cells to kill tumors. Monoclonal antibodies induce the death of tumor cells through several mechanisms, including growth receptor blockade, complement activation, and ADCC [110]. Also, the impact of polymorphisms on the gene encoding CD16 in response to mAb treatment has demonstrated the importance of NK cells in mediating the anti-tumor responses through ADCC. There is a single nucleotide polymorphism in CD16 that results in an amino acid substitution at position 158 (CD16-F158V), and NK cells with the CD16-158V genotype have a higher affinity for IgG1 and IgG3 than those with the CD16-158F genotype, and perform ADCC more efficiently. This polymorphism reinforces ADCC in vivo, such that, for example, patients with lymphoma that are homozygous for CD16-158V show substantially higher response rates after treatment with rituximab than those with CD16-158F polymorphism [111,112,113].

BiKEs and TriKEs (bi- and tri-specific killer engagers) are molecules that act through ADCC by cross-linking epitopes in tumor cells with the CD16 receptor in NK cells [7,80,114]. These molecules have advantages over mAbs because they bind to a different epitope of the CD16 molecule, and results in an NK cell with a more potent ADCC [115,116]. In vitro, the CD16xCD33 BiKE is even capable of overcoming the KIR-mediated inhibitory signal, leading to robust cytokine production and the death of myeloid malignant cells [115,116]. In vivo, treatment with the CD16xCD33 BiKE successfully reversed myeloid-derived suppressor cells’ (MDSCs) immunosuppression of NK cells and induced killing of CD33+ MDSCs and CD33+ myelodysplastic syndrome (MDS) targets [116]. More recently, it has been shown that NK cells treated with a CD16xCD33xIL-15 TriKE proliferate and became activated to overcome dysfunctional NK cells found in MDS [117]. Moreover, IL-15 treatment alone induces the expression of TIGIT, but not when IL-15 is presented in the context of the TriKE [117]. The design of a trifunctional NK cell engager consisting of mAb fragments targeting the activating receptor NKp46 together with a tumor antigen and an Fc fragment to promote ADCC via CD16 has also been described [118]. The authors showed that this trifunctional NK cell engager exhibited superior killing capacity compared with the available therapeutic mAbs in vitro and in vivo [118].

Another strategy is to make tumor cells more susceptible to NK cell-mediated lysis. In this sense, TRAIL, which is expressed in NK cells, triggers apoptosis in TRAILR-positive tumor cells by initiating excision of caspase 8 [119], and it occurs independently of the signals from inhibitory receptors such as CD94/NKG2A and KIR. Exposing tumor cells to proteasome inhibitors such as bortezomib and carfilzomib, which simultaneously positively regulate the expression of TRAILR, make tumor cells more sensitive to NK cell-mediated lysis [120]. Proteasome inhibitors can also sensitize tumor cells to NK cells through positive regulation of NKG2D ligands on the surface of the tumor cells [121].

Other therapeutic strategies consist in the infusion of NK cells into cancer patients. This allows the possibility of manipulating them before infusion. The adoptive transfer of ex vivo activated allogeneic NK cells in the short term can induce clinical responses in patients with AML and in patients with multiple myeloma [77,122]. Many of these clinical trials involve chemotherapy with fludarabine and cyclophosphamide, with or without irradiation as a preparatory regimen to prevent rejection of infused cells, to provide space for persistence and expansion of infused cells and eradicate suppressor cell populations that inhibit NK cell function. Haploidentical NK cells stimulated for a short period of time with high doses of IL-2 before infusion have been used. In addition, the administration of IL-2 after the transfer of adoptive cells is able to further promote the in vivo expansion of infused NK cells, improving objective response rates [77]. More recently, the efficacy and safety of cytokine-induced memory-like (CIML) NK cells have been explored [123]. These CIML NK cells are generated after their exposure for 16–18 hours to a cocktail of IL-12, IL-15, and IL-18. CIML NK cells have increased effector functions (cytotoxicity and cytokine production) after a resting period and a longer half-life [124,125,126,127,128]. Clinical trials have shown its effectiveness in the treatment of patients with an AML refractory to standard treatments [123]. Other methods of adoptive cell therapy involve the ex vivo expansion of NK cells. In contrast to cytokine stimulation for a short period of time, ex vivo expansion allows the use of multiple infusions of highly activated NK cells [80,129,130,131,132]. The development of efficient methods to genetically manipulate NK cells has been seen as a necessity to optimize their persistence in vivo, as well as their location and cytotoxicity against the tumor cells after the adoptive transfer [133,134,135]. Finally, clinical trials are being conducted in which NK cells expressing CAR (chimeric antigen receptor) are administered to patients, after having proven their efficacy in preclinical models [134,135,136,137,138].

## 5. NK Cells and Renal Cell Carcinoma

Renal cell carcinoma (RCC) is the most common type of kidney cancer in adults, representing about 85% of diagnoses. This type of cancer develops in the proximal renal tubules, and about 70% of RCCs are made up of clear cells. Other less common kidney cancers include urothelial carcinoma, sarcoma, lymphoma, and the Wilms tumor, where the latter is the most common kidney cancer in children. 

Several works have reported the presence of NK cells in RCC [139,140,141,142,143] where they may represent a critical component of the anti-tumor response as shown by the association of the NK cell infiltrate with patients’ survival [140,141,144,145,146,147,148]. NK cells also infiltrate RCC lung metastasis, and high NK numbers have been associated with improved survival [145]. Nevertheless, despite the NK cell infiltration, tumors are able to grow, indicating that the tumor microenvironment (TME) negatively affects NK cell functionality. It is well-known that there is an immunosuppressive state in the TME that affects, among others, NK cells [149,150,151,152]. Thus, NK cells infiltrated in clear cell RCC have an altered phenotype and poor degranulation activity compared with circulating NK cells and with those from non-tumor kidney cortices [139,141]. The deficient NK cells are characterized by dampened mitogen-activated protein kinase pathway activation that was dependent on high levels of diacylglycerol kinase (DGK)-α. Restoring NK cell activity was achieved by inhibiting DGK-α and with a brief exposure to IL-2 [141]. Other authors have also shown that NK cells infiltrating clear cell RCC exhibited poor cytotoxic activity against the classical K562 cell target when compared with NK cells from tumor margins and non-tumor tissues. Interestingly, these authors also found that primary tumor cells induced NK cell dysfunction in an exosome-dependent manner [153]. The exosomes were enriched in transforming growth factor (TGF)-β1, which is a well-known mediator that diminishes NK cell-mediated activity in the TME [152,153,154].

A majority of the RCCs have the von Hippel-Lindau (VHL) gene mutated or functionally inactivated [155]. VHL targets the hypoxia-inducible factor (HIF) family of transcription factors, particularly HIF1α and HIF2α, for ubiquitin-mediated degradation in the proteasome [156,157,158]. Therefore, inactivation of VHL leads to an increased expression of HIF. Furthermore, HIF2α has been reported to regulate the expression of a unique set of genes and to be involved in the development of RCC [158,159]. In fact, it has been demonstrated that the human 786-0 RCC cell line with mutated VHL was resistant to the NK cells’ lysis, while the VHL-corrected cell line was susceptible. The NK cell resistance was due to the HIF2α-induced expression of ITPR1 (inositol triphosphate receptor 1), which inhibited NK cell-mediated lysis through a mechanism that involves the induction of autophagy in the target cell after the interaction with NK cells, resulting in granzyme B degradation and target cell survival [156,160]. Nevertheless, the effect of VHL gene mutations on NK cell effector functions is controversial. In this sense, other authors have described that mutations of the VHL gene confer increased susceptibility to NK cell lysis of RCC cell lines and that overexpression of the wild-type VHL gene decreased it, in a mechanism involving the augmented expression of HLA class I molecules [161]. More recently, Trotta et al. have shown that mutated VHL RCC promotes patients’ specific NK cell cytotoxicity. Specifically, they found that IL-2-activated circulating NK cells from RCC patients with mutated VHL exhibited higher degranulation levels and IFNγ production toward a mutated VHL RCC cell line than against a cell line with wild type VHL. Moreover, IL-2-activated NK cells from patients with VHL-mutated RCC displayed higher degranulation levels against autologous RCC cells and a VHL-mutated RCC cell line than against a cell line with wild type VHL [162]. 

There are several therapeutic approaches to treat renal cancer, including surgery, radiofrequency ablation and cryoablation, radiation therapy, targeted therapy, chemotherapy, and immunotherapy (Table 1). Targeted therapy includes anti-angiogenesis therapy aimed to block the vascular endothelial growth factor (VEGF) by means of antibodies (i.e., bevacizumab) and tyrosine kinase inhibitors (TKI), such as axitinib, sunitinib, sorafenib, and so forth. Targeted therapies also include the mTOR inhibitors, everolimus and temsirolimus. Within immunotherapies in renal cancer, some of the current approved treatments include IL-2, IFNα, and immune checkpoint inhibitors (e.g., nivolumab, ipilimumab, pembrolizumab). It is important to note that there are several approved combination therapies. For example, the FDA has approved the combination of TKI axitinib and the checkpoint inhibitor pembrolizumab for the treatment of advanced RCC.

Both TKI and mTOR inhibitors exert antiangiogenic and immunomodulatory functions [163]. In addition to their action on tumor cells, these inhibitors may also inhibit signaling pathways in immune effector cells, such as NK cells. For example, resting and IL-2 activated NK cells exhibited less degranulation and cytokine production when exposed to pharmacological doses of sorafenib, but not sunitinib, in a mechanism involving impaired PI3K and ERK phosphorylation [164]. Other studies have also shown that sunitinib does not impair NK cell function in patients with RCC [165]. On the other hand, sunitinib has been found to induce the expression of MICA, MICB, and ULBP1-3, all of them ligands of the activating NKG2D receptor, on nasopharyngeal cancer cell lines that lead to an increase in their susceptibility to NK cell-mediated cytoxicity [166]. Axitinib is another TKI, which in addition to its direct proapoptotic effect on renal carcinoma cells, is able to increase NKG2D ligands through the DNA damage response (DDR) and, therefore, increasing NK cell recognition and degranulation against a RCC cell line in a reactive oxygen species (ROS)-dependent manner [167]. The combination of sunitinib and immunotherapy has been tested in mouse preclinical models. Specifically, in a mouse model of metastatic RCC, a synergistic anti-tumor response with the combined treatment of sunitinib and an agonistic mAb against the glucocorticoid-induced TNFR related protein (GITR) was shown [168]. Among others, this combined treatment induced a very significant increase in the infiltration and activation of CD8+ T cells and NK cells in liver metastasis [168]. Moreover, cell depletion experiments demonstrated that CD8+ T cells, macrophages, and NK cells infiltrating the metastatic liver contributed to the anti-tumor effect of this combination therapy [168].

In search of prognostic markers that could be associated with the evolution of metastatic RCC patients, levels of Eomes mRNA in peripheral blood were studied before the treatment with sorafenib [169]. Multivariate analysis, including clinical features, identified Eomes mRNA expression levels as a good prognostic marker for progression-free survival and overall survival in these patients treated with sorafenib [169]. Moreover, at protein levels, Eomes was highly expressed on circulating NK cells [169], suggesting that NK cells may play a role in the tumor response in RCC patients treated with sorafenib. On the other hand, in patients with metastatic RCC, the systemic effect of the mTOR inhibitor everolimus was shown to induce immunological alterations in circulating immune cells, including NK cells [170]. Specifically, a significant decrease in the frequency of the CD56^bright^ NK cell subset and the conventional DCs was found, along with an increase in Treg cells and monocyte MDSCs. These data suggest that everolimus may favor immunosuppression and, therefore, that it should be carefully considered in the treatment of these patients [170].

Targeting immune checkpoints, such as PD-1 and CTLA-4, is a success story in the fight against cancer. For example, cancer immunotherapies targeting the PD-1/PD-L1 axis has shown remarkable efficacy in the treatment of many cancers [171,172,173,174,175,176], including RCC [175,176,177,178,179]. Nevertheless, not all patients respond to the therapy, and the identification of treatment response biomarkers is a real need. More importantly, information about blood markers, rather than tumor markers, that are associated with the response to checkpoint inhibition is very scarce. Recently, the changes in blood immune cell subsets and soluble mediators after anti-PD-1 therapy were studied [180]. The authors found that in blood samples from non-small cell lung cancer and RCC patients before treatment, an increased frequency of central memory CD4+ T cells and leukocyte count was associated with a response, while an increased percentage of PD-L1+ NK cells and naïve CD4+ T cells was associated with a lack of response [180]. Considering that it has been proposed that anti-PD-L1 antibodies activate PD-L1+ NK cells to control tumor growth [98] and that the mutated VHL gene induces PD-L1 expression in RCC cells [181], treating patients that have a high frequency of PD-L1+ NK cells with anti-PD-L1 rather than anti-PD-1 antibodies should be considered. Combination therapy of checkpoint inhibitors with other drugs is another reality in the current arsenal for treating RCC patients, and more combination therapies are being tested in preclinical models. For example, the combination of checkpoint inhibitor (CTLA-4) and oncolytic virotherapy has been found to be very complex, and many factors, including viral strains, are critical for the synergistic effects [182]. Once the best condition for the combined administration of anti-CTLA4 antibodies and the oncolytic virus was identified in a mouse model of RCC, it was shown that the synergistic effects of the combined therapy required the participation of CD8+ T cells, NK cells, and IFNγ for the anti-tumor response [182]. Interestingly, HLA-E expression has been reported in RCC tumors [183,184]. However, whether the use of anti-NKG2A Abs could benefit RCC patients remains unexplored.

Tumor-associated antigens are also a focus for the implementation of new therapeutic strategies. Carbonic anhydrase IX (CAIX) is one of the best-characterized antigens associated to RCC [185,186]. Results have shown that human anti-CAIX mAbs induce NK cell-mediated ADCC against RCC cells. Furthermore, engineered anti-CAIX mAbs to enhance the binding affinity to Fc gamma receptors (i.e., CD16) enhanced their ADCC effector function [187]. In an in vivo orthotopic RCC mouse model using human peripheral blood mononuclear cells, it was shown that the anti-CAIX mAbs induced human responses, including NK cell tumor infiltration [187]. Other mAbs are being tested in preclinical models of RCC. In this context, CCR4 is highly expressed in human RCC biopsies and, in a mouse model of RCC, anti-CCR4 mAb was shown to exhibit anti-tumor activity. Interestingly, this anti-CCR4 mAb induced a modification of the immune cell infiltrate in the TME, leading, among others, to an increase in NK cell numbers [188]. More specifically, anti-CCR4 mAb, among other effects, increased the numbers of infiltrating NK cells [188], suggesting that these may participate in tumor elimination following anti-CCR4 mAb administration. Also, it has been shown that SIRPα is highly expressed in human RCC cells, and anti-SIRPα mAbs decreased tumor formation in syngeneic mice [189]. Interestingly, the anti-tumor effect of anti-SIRPα mAbs required not only macrophages, but also NK cells and CD8+ T cells [189].

IL-2 was the first cytokine to be molecularly cloned. The high-affinity IL-2 receptor (IL-2R) is composed of three chains (α, β, and γ), and it is highly expressed in Treg cells, while the intermediate-affinity IL-2R is only composed of the β and γ chains, and is expressed in the majority of T and NK cells [190,191,192]. Soon after the discovery that this cytokine stimulates T and NK cell proliferation and the generation of effector T cells, clinical trials were carried out to evaluate its ability to stimulate anti-tumor responses in patients with renal cancer, melanoma, and other tumors [78,79,85,190]. In fact, high doses of IL-2 were approved by the FDA in 1992 to treat patients with metastatic RCC [78,193]. Evaluating data from a cohort of patients treated with high doses of IL-2, it was found that when the higher-affinity genotypes for FCGR2A, FCGR3A, and FCGR2C were considered together, they were associated with increased tumor shrinkage and prolonged survival [194]. FCGR3A encodes for CD16 expressed on NK cells. On the other hand, in the same cohort of patients, an association of the KIR/KIR-ligand genotype with patient outcomes was not found [195]. IL-2 has also been administered to RCC patients in combination with IFNα, which has been proven to enhance NK cell cytotoxicity and expansion [196,197]. However, several studies have also pointed out that insufficient activation of RCC tumor-infiltrating NK cells contributed to the failure of IL-2 treatment alone or in combination with other agents, such as IFNα [147,198,199].

IL-15 is another cytokine that signals through the IL-2Rβγ, and it is being tested in clinical trials for cancer immunotherapy, including patients with metastatic RCC. A clinical trial has demonstrated acute lymphocyte dynamics with a redistribution of NK cells and CD8+ T cells, followed by hyperproliferation and increased numbers of NK cells (and T cells) after 2–3 days of the infusion. Cell numbers returned to baseline levels around 6 weeks after IL-15 infusion [88]. Another trial using subcutaneous IL-15 administration induced a profound expansion of NK cells, especially the CD56^bright^ subset. In this trial, objective responses were not observed, although some patients had disease stabilization [200] (NCT01727076). Finally, a trial with ALT-803, a complex containing two molecules of mutated IL-15 and two molecules of the IL-15Rα “sushi” domain fused to human IgG1 Fc, also demonstrated expansion and activation of NK cells [201].

Adoptive cell therapy has also been used for the treatment of metastatic RCC. Infusion of activated NK cells in combination with IL-2 [81,202,203], and the adoptive transfer of allogeneic NK cells [77] and NK cell lines, such as NK-92 [204], have been tested [142,205]. Infusions of NK cells have also been combined with other therapeutic procedures. For example, combining allogeneic NK cell therapy with percutaneous cryoablation had a synergistic effect, improving the quality of life of the patients, and also exhibited clinical efficacy [206] (NCT02843607). Also, genetically modified NK cells are being studied for their potential use in the clinic [142]. Given that there are higher concentrations of CXCR2 ligands in tumors compared with the plasma of RCC patients, the properties of NK cells engineered to express CXCR2 were tested [207]. CXCR2 expressing NK cells were able to migrate along a chemokine gradient of RCC tumor supernatants, and this enhanced trafficking resulted in an increased killing of target cells [207]. CAR-engineered NK cells are also being tested in preclinical models. Some examples include the ability of NK-92 cells expressing an ErbB2 (Her2)-specific CAR to reduce lung metastasis in a RCC model [208], how the combination of cabozantinib and NK-92 cells expressing an EGFR-specific CAR exhibit synergistic therapeutic efficacy against the human RCC xenograft model [209], and how bortezomib improves adoptive CAIX-specific CAR-modified NK-92 cell therapy in mouse models of RCC as well [210].

## 6. Conclusions

As summarized in this review, NK cells present several features that make them suitable for fighting against malignant pathologies, and RCC is not an exception. Multiple strategies have been developed to enhance anti-tumor activities of NK cells, and some of them are currently being tested in clinical trials with RCC patients. Therefore, based on current knowledge, we consider that NK cell-based immunotherapies represent a promising tool in the treatment of renal cancers.

## Figures and Tables

**Table 1:** Selected clinical trials on Renal Cell Carcinoma and NK cells (ClinicalTrials.gov).

NCT number	Treatment	NK cell-related analysis	Title of the clinical trial
NCT02843607	Cryosurgery + NK cell infusion	Not specified	Combination of Cryosurgery and NK Immunotherapy for Advanced Kidney Cancer
NCT00328861	Chemotherapy + IL-2 (Aldesleukin) + NK cell infusion	Not specified	Natural Killer Cells Plus IL-2 Following Chemotherapy to Treat Advanced Melanoma or Kidney Cancer
NCT03319459	Group 1: activated NK cell infusion (FATE-NK100) Group 2: activated NK cell infusion (FATE-NK100) + anti-HER-2 (Trastuzumab) Group 3: activated NK cell infusion (FATE-NK100) + anti-EGFR (Cetuximab)	% NK cells	FATE-NK100 as Monotherapy and in Combination With Monoclonal Antibody in Subjects With Advanced Solid Tumors
NCT03841110	Group 1: Lympho-conditioning chemotherapy + iPSC-derived NK cell infusion (FT500) Group 2: Lympho-conditioning chemotherapy + iPSC-derived NK cell infusion (FT500) + anti-PD-1 (Nivolumab or Pembrolizumab) or anti-PD-L1 (Atezolizumab)	iPSC-derived NK cell persistence	FT500 as Monotherapy and in Combination With Immune Checkpoint Inhibitors in Subjects With Advanced Solid Tumors
NCT04106167	iPSC-derived NK cell infusion (FT500)	Not specified	Long-term, Non-interventional, Observational Study Following Treatment With Fate Therapeutics FT500 Cellular Immunotherapy
NCT01727076	IL-15	NK cell effector functions % NK cells	Recombinant Interleukin-15 in Treating Patients With Advanced Melanoma, Kidney Cancer, Non-small Cell Lung Cancer, or Squamous Cell Head and Neck Cancer
NCT01274273	IL-2 (Aldesleukin) + IFNα + anti-VEGF (Bevacizumab)	NK cell assessment	Study of Interleukin-2, Interferon-alpha and Bevacizumab in Metastatic Kidney Cancer
NCT01550367	Autophagy blocking therapy (HC) + IL-2 (Aldesleukin)	% NK cells	Study of Hydroxychloroquine and Aldesleukin in Renal Cell Carcinoma Patients (RCC)
NCT03891485	anti-PD-1 (Nivolumab)	NK cell effector functions	Nivolumab in mRCC Patients: Treg Function, T-cell Access and NK Interactions to Predict and Improve Efficacy
NCT03628859	Group 1: anti-PD-1 (Nivolumab) Group 2: TKI (Axitinib or Cabozantinib) Group 3: mTOR inhibitor (Everolimus)	NK cell effector functions NK cell phenotype	BIOREN (Predictive BIOmarkers in Metastatic RENal Cancer)
NCT01144169	Autophagy blocking therapy (HC) + Surgery	NK cell effector functions NK cell phenotype % NK cells	Study of Hydroxychloroquine Before Surgery in Patients With Primary Renal Cell Carcinoma

Abbreviations: TKI: tyrosine kinase inhibitor; HC: Hydroxychloroquine; iPSC: induced pluripotent stem cell.
